# Metabolic Alkalosis resulting from a Congenital Duodenal Diaphragm

**Published:** 2014-10-20

**Authors:** Passariello A, Maddalena Y, Malamisura M, Rojo S, Aragione N, Iaccarino E, Franco G, Giliberti P

**Affiliations:** 1 Neonatal Intensive Care Unit of Dei Colli Hospital of Naples; 2 Department of Translational Medical Science, University of Naples "Federico II", Naples; 3 Neonatal Intensive Care Unit of Santobono-Pausilipon Hospital of Naples, Italy

**Keywords:** Metabolic alkalosis, Urinary electrolytes concentration, Neonatal surgery

## Abstract

Duodenal diaphragm is an unusual cause of upper intestinal obstruction. We present here a neonate with duodenal diaphragm who presented with features of metabolic alkalosis. Further, an algorithm of management of metabolic alkalosis in a newborn is suggested.

## INTRODUCTION

Duodenal diaphragm (DD) is an unusual cause of incomplete high intestinal obstruction. Postprandial vomiting, sometimes bilious, is often the main presenting symptom. The diagnosis of high intestinal obstruction is generally made on plain abdominal X-ray; however confirmation may require barium examination. Early diagnosis and surgical treatment are required to avoid any significant morbidity. We report DD in a neonate who presented with metabolic alkalosis (MA) that is undoubtedly a rare presentation. We also suggest a diagnostic algorithm that would help in evaluate MA in the neonates.

## CASE REPORT

A 14-day-old female, born at 40 weeks’ gestation with a birth weight of 2.97 Kg, referred from another hospital to us with persistent bile-stained vomiting and metabolic imbalance. She was doing well (other than frequent regurgitation of breast milk) and gaining weight for first 12 days of life, when she started vomiting almost all feeds. At the sub-intensive care unit, the following biochemical abnormalities were noted; pH 7.73, bicarbonate 35.8mEq/L, pCO2 27 mmHg with Na+ 124 mEq/L, Cl- 72 mEq/L, and creatinine 0.70 mg/dL. The abdominal ultrasound scan did not reveal any hypertrophic pyloric stenosis. Upper gastro-intestinal contrast series was essentially normal other than significantly delayed emptying of stomach. 

She was transferred to our Neonatal Intensive Care Unit on the fourteenth day of life for persistent MA. She was severely dehydrated and her serum biochemistry showed pH 7.65, bicarbonates 44 mEq/L, Na+ 129 mEq/L, K+ 2.7 mEq/L, Cl-79 mEq/L. The abdomen was not distended, but the nasogastric tube was draining bile-stained fluid. Abdominal ultrasound scan showed modest ascitic effusion in sub-hepatic region and aimless peristalsis between the third and fourth duodenal portion. 

We proceeded initially to exclude the non-surgical causes of neonatal persistent vomiting with specific tests (Fig. 1). She had persistently low urinary electrolytes concentrations with normal serum electrolytes values. Upper gastrointestinal series was repeated that showed severe delayed gastric emptying (presence of contrast in stomach after 6 hour) and markedly dilated proximal duodenum with hypotonic duodenal bulb. Incomplete obstruction at duodenal ‘C’ was also observed.

The patient subsequently underwent laparotomy wherein duodenal diaphragm was excised. The patient made an uneventful recovery. 

The most significant abnormality in our patient at presentation was metabolic alkalosis. It is a relatively common clinical problem, resulting from hydrogen loss, hydrogen movement into the cells, alkali administration, or volume contraction around a relatively constant amount of extracellular bicarbonate. Loss of hydrogen ions from the gastrointestinal tract or in the urine are the most common causes; it is usually accompanied by hypokalemia [1]. The estimation of urine chloride concentration is of important diagnostic value (Fig. 1). Our patient presented urine chloride of 10 mEq/L despite infusional therapy, compatible with vomiting due to intestinal obstruction.

**Figure:**
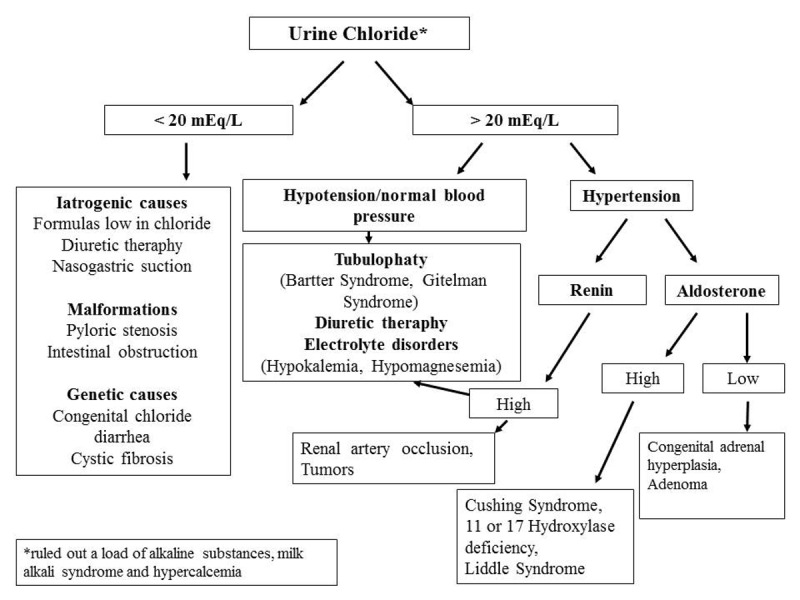
Figure 1: Diagnostic algorithm of metabolic alkalosis based on urine chloride.

The differential diagnoses for persistent vomiting such as in the index case include gastroesophageal reflux disease, milk protein induced enteritis, adrenal insufficiency, inborn errors of metabolism, pyloric stenosis, intestinal obstruction, malrotation, Hirschsprung’s disease [2]. Sodium wasting is a common finding in the first few days and occasionally later in patients with vomiting because the hypovolemic drive to retain sodium can be counteracted by the excretion of sodium and potassium bicarbonate when the serum bicarbonate level has risen rapidly and exceeds the renal threshold [1].

Significant bicarbonaturia is typically characterized by a urine pH above 7.0, while bicarbonate excretion is low at a urine pH below 6.5 [1].

Congenital duodenal obstruction is an uncommon condition, with an incidence of 1 in 4.000-15.000 live births [3]. During the 8th-10th weeks of intrauterine life, the duodenal lumen is obliterated by rapidly growing epithelium, which then recanalizes in the 12th week. Failure of recanalization or arrest in duodenal growth results in the development of duodenal atresia, stenosis, or diaphragm with or without an aperture [4]. The majority of DD are situated between the third and fourth portions of the duodenum, near the ampulla of Vater [4]. Vomiting is a very common symptom, it often causes a MA with hypochloraemia, as in our patient. Food refusal with failure to thrive is also expired by the patients [5]. Sometimes DD can occur with blurred symptoms, especially if there is a partial obstruction and it can be discovered later in the life [5,6]. Furthermore the dilatation of the stomach, caused by duodenal obstruction, may produce hypergastrinemia and peptic ulceration [4]. It is possible to explain adult manifestations by a progressive loss of compensatory peristaltic action to overcome the small duodenal hole [6]. 

Intrinsic duodenal obstruction may be associated with other conditions like prematurity, Down’s syndrome, situs inversus and coexistent anomalies [7]. A recent study has revealed a correlation between a “de novo” inversion of chromosome 9 (inv9) (p11q13) and some congenital anomalies, including the DD [8]. 

Early diagnosis and surgical treatment are required to significantly prevent the infant morbidity. The diagnosis can be made by a plain abdominal X-ray that shows the classic “double-bubble” appearance; barium upper gastrointestinal examination is more useful in those with partial duodenal obstruction secondary to a duodenal web with a central aperture [9].

Upper gastrointestinal series and endoscopic study is useful to differentiate DD from annular pancreas, in fact the wall of the diaphragm appears as a lucent line when barium passes through the eccentric hole [4].

The intervention is necessary as soon as the diagnosis is obtained. Surgical treatment in children and endoscopic membranectomy in adult are preferred [3]. 

In conclusion, duodenal diaphragm is a rare cause of intestinal obstruction; recognition and surgical treatment are crucial.

## Footnotes

**Source of Support:** Nil

**Conflict of Interest:** None

